# Oxytocin neurons in the anterior and posterior paraventricular nucleus have distinct behavioral functions and electrophysiological profiles

**DOI:** 10.1038/s41386-026-02352-y

**Published:** 2026-01-17

**Authors:** Audrey N. Chrisman, Chiho Sugimoto, Hanna Butler-Struben, Vanessa A. Minie, Andrew L. Eagle, Daniela Anderson, Natalia Duque-Wilckens, Ashley Ramos, Yasmine I. Lewis, Sinéad C. Archdeacon, Alfred J. Robison, Brian C. Trainor

**Affiliations:** 1 Neuroscience Graduate Group, University of California, Davis, CA USA; 2 Department of Physiology, Michigan State University, East Lansing, MI USA; 3 Animal Behavior Graduate Group, University of California, Davis, CA USA; 4 Department of Psychology, University of California, Davis, CA USA; 5 Department of Neuroscience, University of Texas, Dallas, TX USA; 6 Department of Biology, North Carolina State University, Raleigh, NC USA

**Keywords:** Cellular neuroscience, Social neuroscience, Neuronal physiology

## Abstract

Oxytocin is a neuropeptide that can promote or inhibit affiliative social behaviors. Recent evidence suggests that these diverse effects are mediated by distinct oxytocin receptor-expressing neurons. An outstanding question is whether these behavioral effects are also driven by distinct or overlapping populations of oxytocin-producing neurons. The paraventricular nucleus (PVN) of the hypothalamus is a major source of oxytocin and sends projections to the mesolimbic dopamine system and extended amygdala. Previous work found that social defeat stress increased oxytocin neuron activity in the anterior PVN (aPVN) but not posterior PVN (pPVN). We reduced oxytocin synthesis with antisense morpholino oligonucleotides in either anterior or posterior PVN in California mice (*Peromyscus californicus*), a strong model system for studying effects of social stress on brain function and behavior. Antisense morpholinos in aPVN had no effect on behavior in unstressed females but increased social approach and reduced social vigilance in females exposed to social defeat stress. In pPVN, antisense morpholinos reduced social approach in unstressed male and female California mice. We then used *Oxt*^Cre^ mice to compare electrophysiological profiles of oxytocin in aPVN and pPVN with a population of oxytocin neurons in the bed nucleus of the stria terminalis (BNST). Oxytocin neurons in aPVN and BNST had higher post-synaptic events and responded more strongly to current injections than oxytocin neurons in pPVN, though they had similar excitatory and inhibitory input balance at the observed resting membrane potential. These findings shed light on to functional and physiological heterogeneity of PVN oxytocin neurons. Our results suggest that context-dependent behavioral effects of oxytocin are mediated by different populations of oxytocin neurons.

## Introduction

Oxytocin is widely known to promote affiliative social behaviors [1–3] and has generated interest as a potential therapeutic [4]. A challenge for using oxytocin-based therapeutics is that in humans [5, 6] and animals [7, 8] oxytocin can sometimes increase anxiety-related behaviors. The social salience hypothesis provides a potential explanation for these observations by positing that oxytocin enhances the salience of social cues across contexts [9, 10]. In affiliative contexts, oxytocin facilitates social approach [11, 12], while in agonistic contexts oxytocin can drive avoidance behaviors [13, 14]. These effects are mediated by different populations of oxytocin receptors [15–17]. Oxytocin receptor antagonists infused into the nucleus accumbens (NAc) reduced social approach in non-stressed California mice [18] and impaired pair bond formation in prairie voles [19]. In contrast, oxytocin receptor agonists infused into the anteromedial bed nucleus of the stria terminalis (BNST) reduced social approach in California mice [20, 21]. While distinct populations of oxytocin receptor neurons have different behavioral effects, it’s less clear whether different populations of oxytocin neurons modulate social approach and avoidance. Calcium imaging of individual oxytocin neurons showed that some oxytocin neurons in the paraventricular nucleus (PVN) are activated by social stimuli while others are inhibited [22]. Oxytocin neurons in the BNST are activated when animals engage in social avoidance [23, 24], and oxytocin knockdown within the BNST increased social approach [20]. It is unknown if BNST oxytocin neurons are unique in promoting social avoidance responses.

Oxytocin neurons in the PVN project to numerous brain regions that impact social behavior, such as the NAc and BNST [25, 26]. Oxytocin neurons are traditionally classified as magnocellular or parvocellular [27], but this binary categorization may be too simplistic [28]. An analysis of morpho-electric properties of PVN oxytocin neurons in C57Bl6/J mice [29] revealed some magnocellular neurons in the posterior PVN (pPVN) are more similar to pPVN parvocellular neurons than to magnocellular neurons in anterior PVN (aPVN). Other data suggests functional differences between oxytocin neurons in aPVN vs pPVN. In C57BL6/J mice, oxytocin neurons that promote social learning are clustered in the pPVN [30, 31]. Conversely, female California mice (*Peromyscus californicus*) that exhibited stress-induced social avoidance had increased oxytocin/c-fos colocalizations in the aPVN but not pPVN [24]. These data suggest that distinct populations of oxytocin neurons within the PVN promote social approach versus avoidance.

To test this hypothesis, we used an antisense morpholino knockdown strategy to selectively inhibit oxytocin production in the aPVN or pPVN in California mice. California mice are territorial and monogamous rodents in which males and females defend joint territories, making this species ideal for studying the impact of social stress in both sexes [32]. Social defeat stress induces lasting social vigilance and reductions in social approach in female but not male California mice [33, 34]. In the aPVN, where oxytocin neurons are more reactive in female California mice after stress, we tested whether oxytocin knockdown affected behavior in control or stressed females. In the pPVN, where defeat stress had no long-lasting effects on oxytocin neural reactivity, we examined how oxytocin knockdown affected behavior in stress-naïve males and females. We complemented these studies with electrophysiological analyses of oxytocin neurons in the aPVN, pPVN, and BNST from stress naïve *Oxt*^Cre^ C57Bl/6 J mice [35, 36]. Our results suggest that the electrophysiological and functional properties of aPVN oxytocin neurons more closely resemble BNST oxytocin neurons than pPVN neurons.

## Methods

### Animals

For behavioral experiments, California mice (*Peromyscus californicus)* were bred and maintained in a colony at UC Davis on a 16 L:8D light-dark cycle with *ad libitum* access to food (Harlan Teklad) and water. All animals were at least 90 days old, and all behavioral assessments were conducted during the dark phase under dim red light (3 lux). For electrophysiology experiments, *Oxt*-IRES Cre mice (Oxt^Cre^, Strain #024234) were purchased from Jackson Labs and crossed with Cre-inducible Rosa^eGFP-L10a^ mice (Gift of Dr. Gina Leinninger at Michigan State University). Methods for slice electrophysiological recordings are described in Supplementary Methods. All procedures followed NIH guidelines and were approved by UC Davis and Michigan State University Institutional Animal Care and Use Committees.

### Social defeat stress

Female California mice were randomly assigned to social defeat stress or a control handling condition [33, 37]. In the social defeat stress group, mice were placed in the home cage of a highly aggressive same-sex counterpart and were either exposed to 7 bites from the resident or remained in the cage for 7 min, whichever occurred first. Mice assigned to the control group were placed into an empty cage for 7 min. Social defeat sessions and control handling sessions occurred for three consecutive days. After each episode, all mice were returned to the home cage.

### Morpholino inhibition of oxytocin synthesis

**Fig. 1:**
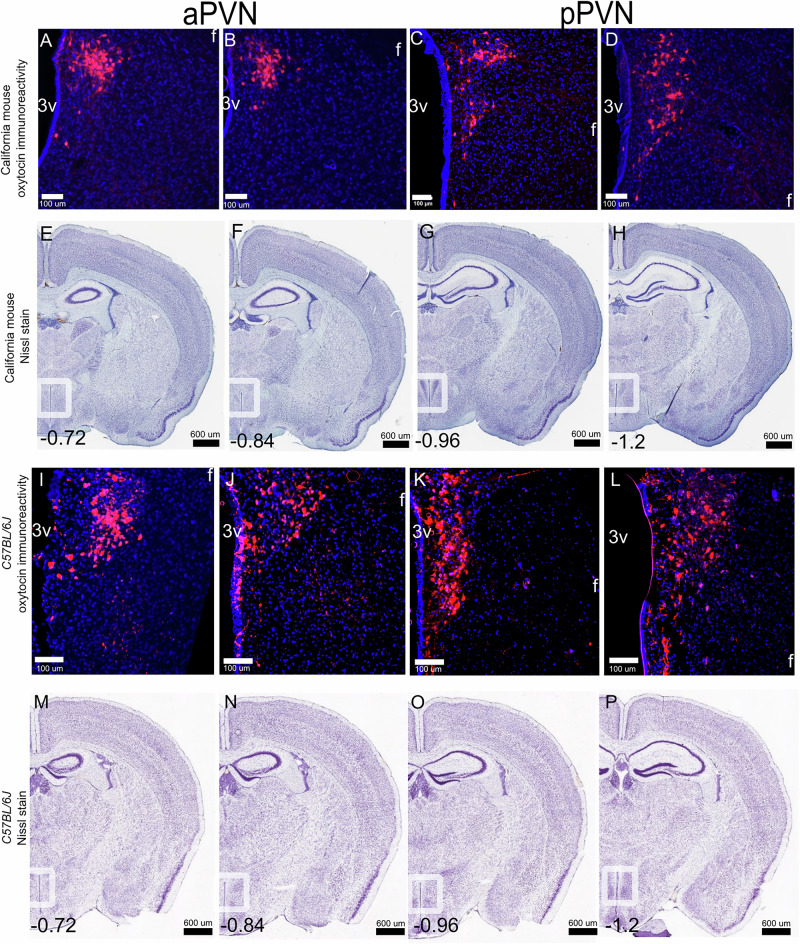
Oxytocin neurons in the paraventricular nucleus. Distribution of oxytocin neurons (red) across the anterior-posterior extent of the paraventricular nucleus in California mice (**A**–**D**) and Mus musculus (**I**–**L**) with corresponding Nissl images from the Brainmaps California mouse brain atlas (**E**-**H**) (brainmaps.org) and Allen mouse brain atlas (**M**–**P**).

A morpholino stock solution (Genetools) was created using artificial cerebrospinal fluid to create a 150 pM solution. The antisense sequence targeting oxytocin mRNA, previously validated in the BNST [20], was 5′-TTG GTG TTC TGA GTC CTC GAT CC-3′. The missense control sequence was 5′-TTC GTC TTC TGA CTC CTC CAT GC-3′. California mice were randomly assigned to receive a 0.2 μL infusion of either antisense or missense solution. Injections were made either into the aPVN (Fig. 1, coordinates: anterior-posterior: –0.8, medial-lateral: ±0.3, dorsal-ventral: −6.1) or pPVN (Fig. 1, coordinates: anterior-posterior: –1.2, medial-lateral: ±0.7, dorsal-ventral: −5.1) using a sterile glass pipette. Carprofen (5 mg/kg) was administered subcutaneously during surgery and once daily for the next 3 days. Six days after surgery, behavior was assessed in the social interaction test. Two mice were excluded from the study due to bleeding during morpholino injection.

### Social interaction test

The social interaction test consisted of three consecutive three-minute phases: open field, habituation, and interaction. In the open field phase, the focal mouse roamed freely in the 89 cm × 63 cm × 60 cm arena. In the acclimation phase, a 22 cm × 15 cm × 15 cm wire cage was introduced into the back of the arena to familiarize the mouse with the new structure before introducing the target mouse. In the interaction phase, a novel conspecific target mouse of the same sex was placed into the wire cage inside the arena, allowing the focal mouse to interact with the caged target mouse. An overhead camera and ANY-maze (Stoelting) were used to record time spent in the interaction zone (within one body length of the wire cage), time spent in the center, and total distance. Vigilance behavior in videos of the interaction phase was manually scored by the time spent outside the interaction zone and with the head oriented to the target cage. The time spent engaging in vigilance out of the three-minute trial was quantified using BORIS [38].

### Quantification of oxytocin knockdown

Photomicrographs of the tissue were captured using a Keyence BZX-800. We used ImageJ (NIH, Bethesda, MD) to analyze percent staining for each image. An observer who was unaware of treatment group assignment counted cell bodies with positive oxytocin staining. Anterior PVN was defined as –0.8 to –0.99 Bregma and pPVN was defined as –1.0 to –1.2 Bregma. The number of oxytocin cells was averaged from 2 to 3 slices of aPVN or pPVN. Slices were anatomically labeled by position of nearby fornix (Fig. 1). Boxes for anterior (650 × 560 mm) and posterior PVN (727 × 1090 mm) were used for cell counts. Consistent with our previous use of morpholinos [20], Nissl stains indicated no evidence of cytotoxicity (Fig. S1).

### Statistical analysis

Cell counts and data from the social interaction tests were analyzed using two-way ANOVA (stress and morpholino for aPVN; sex and morpholino for pPVN) followed by planned comparisons between antisense and missense groups. Planned comparisons were used to test the effects of antisense treatment versus missense. Kruskal-Wallis tests were used to analyze social vigilance data that had heterogenous variability across groups. One-way ANOVA was to analyze sEPSC amplitude, frequency, capacitance, membrane resistance, and resting membrane potential across different cell types. A two-way mixed effects model (cell type and current injection) was used to analyze spike frequency data.

## Results

### Oxytocin neurons in anterior and posterior PVN

Coronal sections across the anterior-posterior extent of the PVN were used to characterize the distribution of oxytocin neurons in anterior versus posterior PVN. In both California mice (Fig. 1A, B, E, F) and C57lBl6/J (Fig. 1I, J, M, N), aPVN oxytocin-positive neurons are located below the dorsal extent of the 3rd ventricle (3 v), and the fornix is positioned lateral and dorsal to these cells. In California mice (Fig. 1C, D, G, H) and C57Bl6/J (Fig. 1K, L, 1O, P), pPVN oxytocin neurons are positioned adjacent to the dorsal border of the 3 v and the fornix is lateral and ventral (Fig. 1C, D, K, L). The distribution of GFP-positive oxytocin neurons in Oxt^Cre^/Rosa^L10eGFP^ mice mirrored the distribution of oxytocin-positive neurons in wild-type C57Bl6/J mice Fig. S2).

### Knockdown of oxytocin in the anterior PVN

**Fig. 2:**
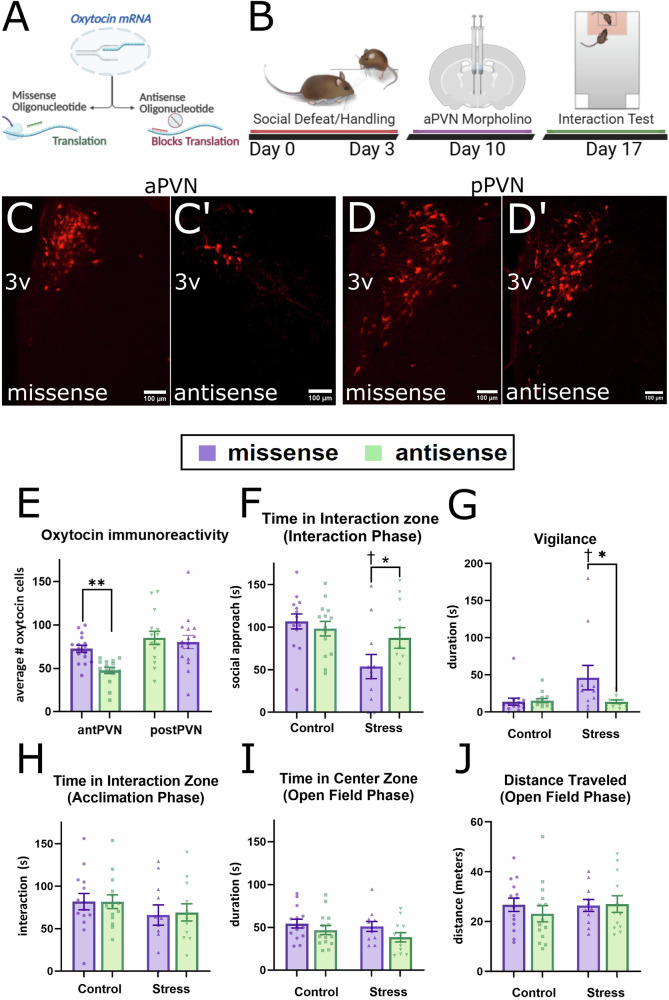
Effects of targeted knockdown of oxytocin via morpholinos in the anterior PVN of socially defeated or control handled female California mice. **A** Morpholinos block translation of oxytocin mRNA. **B** Experimental timeline. **C** Representative photomicrographs of oxytocin immunostaining in the anterior PVN of missense-treated and **C**’ antisense-treated mice. **D** Representative photomicrographs of oxytocin immunostaining in the posterior PVN of missense-treated and **D**’ antisense-treated mice. **E** Oxytocin immunoreactivity in the anterior and posterior PVN of missense and antisense-treated mice. **F** Effects of morpholino treatment on time spent in the interaction zone with a novel conspecific in stressed and control females, **G** social vigilance behavior during the social interaction test, **H** time spent in the interaction zone with a novel empty cage, **I** time spent in the center zone during an open field test, and **J** distance traveled during an open field test. **p < 0.005 main effect of antisense treatment. *p = 0.05 vs stressed missense. † p < 0.05 vs control missense.

Previous work showed that stress increased the reactivity of aPVN oxytocin neurons in female but not male California mice, so we used morpholinos to reduce oxytocin production in aPVN of female California mice (Fig. 2A, B). Antisense injected into aPVN reduced the number of oxytocin-immunoreactive neurons in aPVN (Figs. 2C–E and S3 and Table S1; main effect of treatment, F_1,58_ = 13.97, p = 0.004) but not pPVN (Figs. 2C–E and S3, Table S1). Although we did not have a fluorophore to track the diffusion of morpholinos, these data provide strong evidence that this approach yielded region-specific knockdown. The effects of oxytocin knockdown on social approach were stress-dependent (Fig. 2F and Table S2, stress*treatment F_1,47_ = 3.83, p = 0.05). In missense-treated females, social approach was significantly lower in stressed mice (Fig.2F, planned comparison p < 0.001, Cohen’s d = 1.19). Stressed females treated with antisense showed more social approach than stressed females treated with missense (Fig. 2F, planned comparison p = 0.05, d = 0.76). Similar patterns were observed for social vigilance (Fig. 2G, Kruskal-Wallis p < 0.05, d = 0.71). In missense-treated females, stressed mice showed more vigilance than controls (Fig. 2G, Mann-Whitney p < 0.05). Stressed females treated with antisense had lower levels of social vigilance compared to stressed females treated with missense (Fig. 2G, Mann-Whitney p < 0.05, d = 0.76). Thus, while our knockdown approach reduced oxytocin immunoreactivity by about two-fold, this reduction was sufficient to reduce the impact of social stress on behavior. There were no differences in time spent interacting with an empty cage (Fig. 2H), time spent in the center of the arena during the open field phase (Fig. 2I), or general locomotion (Fig. 2J). These data suggest that oxytocin neurons in the aPVN have similar functions to oxytocin neurons in the BNST.

### Knockdown of oxytocin in the posterior PVN

**Fig. 3:**
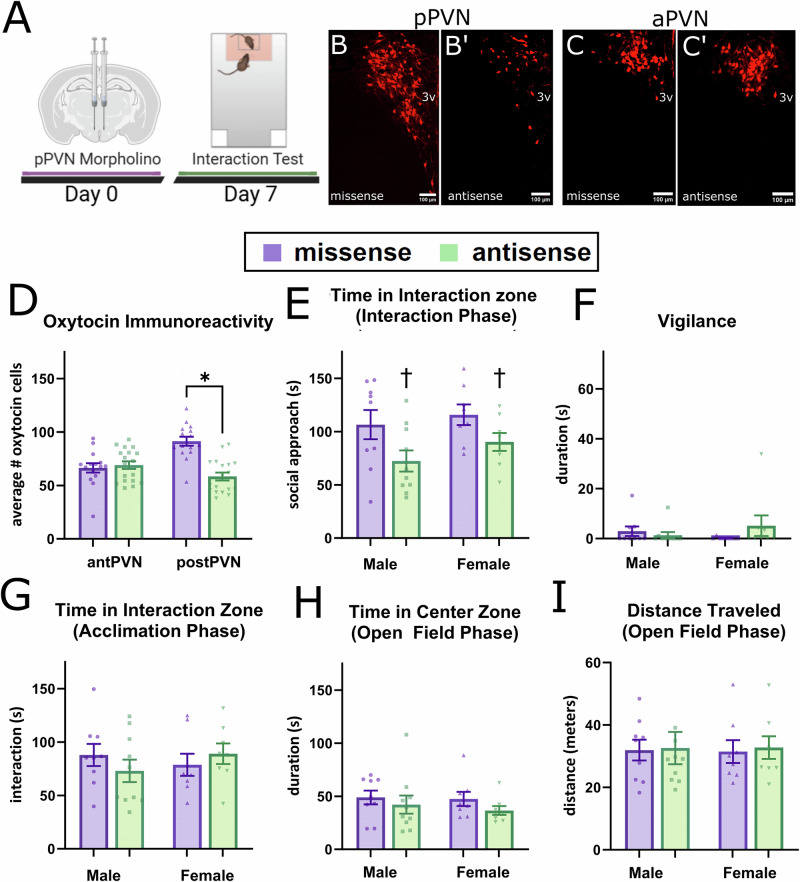
Effects of targeted knockdown of oxytocin via morpholinos in the posterior PVN of male and female California mice. **A** Experimental timeline. **B** Representative photomicrographs of oxytocin immunostaining in the posterior PVN of missense-treated and **B**’ antisense-treated mice. **C** Representative photomicrographs of oxytocin immunostaining in the anterior PVN of missense-treated and **C**’ antisense-treated mice. **D** Oxytocin immunoreactivity in the anterior and posterior PVN of missense and antisense-treated mice. **E** Effects of morpholino treatment on time spent in the interaction zone with a novel conspecific in male and female mice, **F** social vigilance behavior during the social interaction test, **G** time spent in the interaction zone with a novel empty cage, **H** time spent in the center zone during an open field test, and **I** distance traveled during an open field test. N = 11–15 per group. *p < 0.05 vs main effect of antisense treatment. † p < 0.01 vs main effect of antisense treatment.

In the California mouse pPVN, oxytocin morpholino treatment had different behavioral effects (Fig. 3A). Antisense injected into the pPVN reduced the number of oxytocin-immunoreactive neurons in pPVN (Figs. 3B, C, D  and S3, Tables S1 and S3; main effect of treatment, F_1,31_ = 7.38, p = 0.01) but not aPVN (Figs. 3D and S3). In unstressed males and females, antisense morpholino treatment reduced social approach (Fig. 3E; main effect F_1,31_ = 7,54, p = 0.01). There were no significant sex differences or sex*treatment interactions. Morpholino treatment did not affect social vigilance (Fig. 3F), interaction with the empty cage during acclimation (Fig. 3G), time spent in the center of the arena (Fig. 3H), or general locomotion (Fig. 3I) during an open field test.

### Electrophysiological characterization of oxytocin neurons

**Fig. 4:**
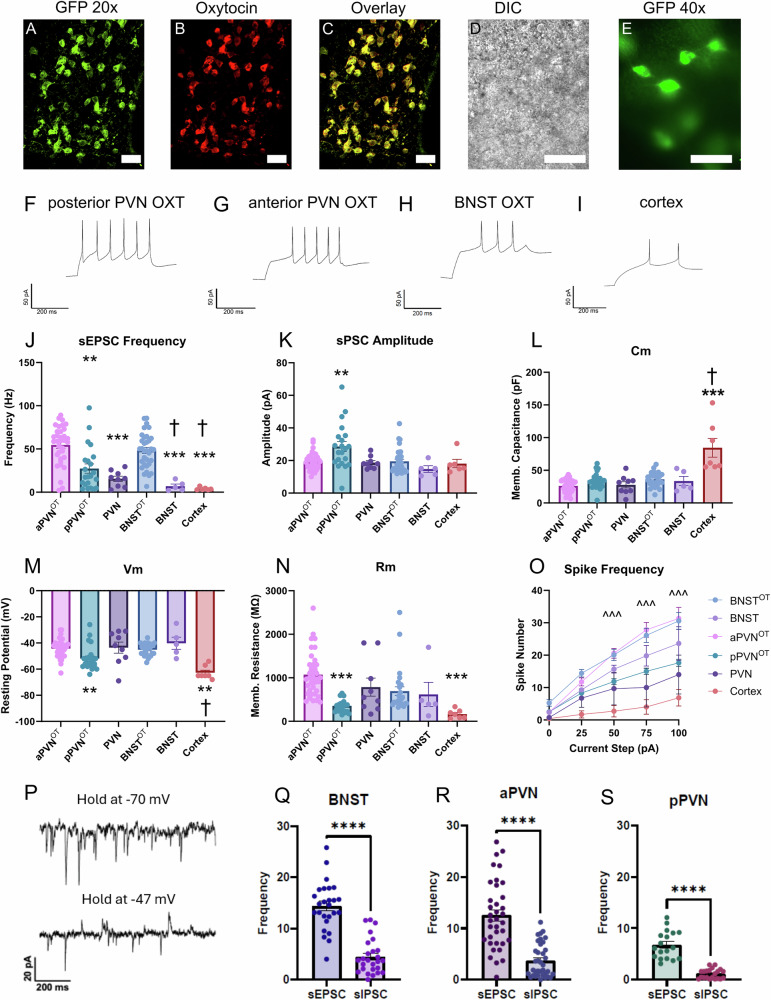
Electrophysiological characteristics of oxytocin and non-oxytocin neurons in the BNST and PVN of *Oxt*^Cre^/Rosa^L10eGFP^ mice. Representative images of: **A** GFP (20x), **B** Oxytocin immunostaining (20x), **C** GFP-Oxytocin overlay (20x), **D** Differential interference contrast (DIC) image (40×), **E** GFP (40x). Representative current-clamp recordings from: **F** posterior PVN oxytocin neurons (pPVN^OT^), **G** anterior PVN oxytocin neurons (aPVN^OT^), **H** BNST oxytocin neurons (BNST^OT^), and **I** cortical neurons. Electrophysiological properties across groups are quantified for: **J** sPSC frequency, **K** sPSC amplitude, **L** membrane capacitance, **M** resting membrane voltage, **N** membrane resistance, **O** spike frequency in response to 0–150 pA current injection. **P** Example current traces showing that oxytocin neurons show both upward and downward events when held at –47 mV. **Q** Frequency of sEPSC and sIPSC in the oxytocin neurons of the BNST, **R** aPVN, and **S** pPVN. **p < 0.01 vs aPVN, ***p < 0.01 vs aPVN^OT^, ****p < 0.001 vs sIPSC † p < 0.01 vs BNST^OT^ ^^^ p < 0.001 aPVN^OT^ vs pPVN^OT^. For cell propagation analyses, a total of 111 cells (64 male, 47 female) were recorded from 35 mice (19 male, 16 female). For sPSC analyses, a total of 133 cells (77 male, 56 female) were recorded from 32 mice (16 male, 16 female). For spike frequency analyses, a total of 92 cells (49 male, 43 female) were recorded from 28 mice (15 male, 13 female).

To characterize intrinsic electrophysiology properties of oxytocin neurons across the PVN and BNST, we performed whole-cell patch clamp recordings from GFP-containing oxytocin and GFP-negative non-oxytocin neurons in *Oxt*^Cre^ mice. There was selective expression of GFP (Fig. 4A) in oxytocin-positive neurons across the PVN (Figs. 4A, B, C) and S4). Interestingly, oxytocin neurons in the pPVN had depolarization kinetics similar to parvocellular oxytocin neurons in Wistar rats [39], as they lacked the transient outward rectification (Fig. 4F) previously observed in magnocellular oxytocin neurons [40]. This outward rectification was observed in aPVN (Fig. 4G) and BNST (Fig. 4H) oxytocin neurons. Cortical neurons (Fig. 4I) and non-oxytocin neurons did not exhibit transient outward rectification.

The frequency of spontaneous postsynaptic currents (sPSCs) differed across brain regions and cell types (Fig. 4J, F_5,103_ = 15, p < 0.0001). Oxytocin neurons in the pPVN (pPVN^OT^) had significantly lower sPSC frequencies than oxytocin neurons in the aPVN (aPVN^OT^; planned comparison p < 0.0001). Anterior PVN oxytocin neurons also had higher sPSC frequencies than non-oxytocin PVN neurons (p < 0.001). These data suggest that oxytocin neurons in the aPVN have more robust synaptic input than pPVN oxytocin neurons. Similarly, BNST oxytocin (BNST^OT^) neurons had higher sPSC frequencies compared to non-oxytocin BNST neurons (p < 0.01). In this respect, BNST and aPVN oxytocin neurons appear to share strong synaptic input or regulation. Cortical neurons recorded on the same slices as PVN neurons had lower sPSC frequencies than aPVN oxytocin (p < 0.01) or BNST oxytocin (p < 0.01) neurons. The amplitude of sPSCs was also recorded across the same six populations, revealing significant differences in the size of individual synaptic events (Fig. 4K, F_5,102_ = 5.08, p = 0.001). Notably, pPVN oxytocin neurons had larger sPSC amplitudes compared to aPVN oxytocin neurons and BNST oxytocin neurons (p < 0.01), suggesting that pPVN oxytocin neurons may receive stronger individual synaptic inputs.

We also assessed whole-cell membrane capacitance across populations. Cortical neurons had significantly higher Cm compared to aPVN oxytocin (Fig. 4L, p < 0.0001) and BNST oxytocin neurons (p < 0.0001). This is consistent with the larger somatodendritic size of cortical pyramidal neurons [41]. Resting membrane potential recordings also significantly varied across cell types (Fig. 4M, F_5,97_ = 9.71, p < 0.001). Within the PVN, posterior oxytocin neurons were more hyperpolarized than anterior oxytocin neurons (p < 0.001). Within the BNST, there were no differences between oxytocin and non-oxytocin neurons. Cortical neurons were the most hyperpolarized as previously observed [42]. There were also cell type differences in membrane resistance (Fig. 4N, F_5,101_ = 9.75, p < 0.0001). Oxytocin neurons in pPVN had lower membrane resistance than aPVN (p < 0.0001), while non-oxytocin PVN neurons were intermediate, suggesting that aPVN oxytocin neurons have reduced leak conductance and may be more sensitive to synaptic input. In the BNST, membrane resistance was comparable between oxytocin and non-oxytocin neurons. Firing responses to stepwise current injection (0–100 pA) also revealed differences between the cell types (Fig. 4O, F_4,81_ = 22.49, p < 0.001). Oxytocin neurons in the aPVN and BNST exhibited higher spike frequencies at all steps above 50 pA (cell type × current F_24,358_ = 8.88, p < 0.001) compared to pPVN oxytocin neurons and non-oxytocin PVN neurons. Cortical neurons had the lowest overall excitability, replicating previous recordings [43]. Lastly, oxytocin neurons were held at –47 mV, closer to the reversal potential for chloride ions at physiological conditions [44] and to the resting membrane potential for these cells. Both downward (sEPSCs) and upward (sIPSCs) were observed at this voltage in all cells (Fig. 4P). There were significantly more sEPSC events than sIPSC events in BNST (Fig. 4Q), aPVN (Fig. 4R), and pPVN (Fig. 4S) (all p < 0.001), with pPVN receiving fewer sEPSCs and sIPSCs. No sex differences were found in recordings (Fig. S4), which is not surprising based on our previous c-fos studies which observe no sex differences in unstressed California mice. Overall, our electrophysiological recordings suggest regional differences of intrinsic excitability, with aPVN and BNST oxytocin neurons sharing a distinct, more excitable phenotype than pPVN oxytocin neurons.

## Discussion

There is growing evidence for heterogeneity in PVN oxytocin neurons, and we defined key anatomical distinctions within the PVN that help to explain how oxytocin produced in the PVN can drive opposing social behaviors. In California mice, we observed that oxytocin in the aPVN facilitates stress-induced social avoidance and vigilance in female California mice, similar to BNST oxytocin neurons [20]. In contrast, oxytocin produced in pPVN facilitates social approach in male and female California mice, similar to studies in other species showing that oxytocin promotes affiliative behaviors [30, 35]. In *Oxt*^Cre^ mice, we observed that the electrophysiological properties of aPVN oxytocin neurons more closely resembled BNST oxytocin neurons as opposed to adjacent pPVN oxytocin neurons. Although the behavioral and physiological data were collected in different species, both data sets indicate functional heterogeneity within PVN oxytocin neurons across the anterior-posterior axis. Thus, while we can’t directly integrate data across the two species, both datasets suggest that aPVN oxytocin neurons share more physiological and functional properties with BNST oxytocin neurons versus pPVN oxytocin neurons. Our results suggest that distinct populations of oxytocin-producing cells are responsible for promoting either social approach or aversion.

### Oxytocin produced in the anterior PVN promotes avoidance and vigilance

In stressed female California mice, oxytocin neurons in the aPVN are more reactive during a social interaction test than in control females [24]. Our knockdown results suggest that aPVN oxytocin neurons contribute to stress-induced social avoidance and vigilance. Others have also reported that aPVN oxytocin neurons do not promote affiliative responses. In wild house mice, aggressive encounters increased oxytocin/c-fos colocalizations in aPVN oxytocin neurons in males and females [14]. Ablation of aPVN oxytocin neurons decreased female aggression, while optogenetic stimulation increased aggression and arousal in females. Although oxytocin is usually considered to promote affiliation [45], it can also promote avoidance of unfamiliar individuals across many different species [7]. Our results suggest that social avoidance is mediated primarily by aPVN oxytocin neurons.

Although aPVN and pPVN differ in glutamatergic forebrain inputs [46], less is known about what these populations of oxytocin neurons are encoding. It is possible aPVN oxytocin neurons project to the anteromedial BNST where oxytocin receptor activation promotes social avoidance and vigilance [21, 47]. This would be consistent with previous reports showing PVN oxytocin fibers in the BNST [25]. Future studies are needed to determine if projections of aPVN oxytocin neurons are distinct from pPVN oxytocin neurons. The mechanisms through which stress increases the excitability of aPVN oxytocin neurons are unknown. Social stress exposure increases the reactivity of dorsal raphe serotonin neurons [48, 49] and activation of serotonin receptors increases burst firing in PVN oxytocin neurons to stimulate oxytocin release [50]. Further study is needed to determine if changes in reactivity are due to changes in synaptic inputs or stress-induced changes in intrinsic excitability. Another possibility is that oxytocin knockdown might trigger compensatory changes in vasopressin, which can have important effects on social approach in male [51, 52] and female [51] rodents. An intriguing possibility is that increased vasopressin release in the BNST could activate V1a receptors, which promote social approach in males and females [53]. Future studies on male aPVN oxytocin neurons are warranted as well. Although social defeat does not have long-term effects on social approach in male California mice, defeat stress does acutely (within 15 min) induce social avoidance and vigilance in both males and females [54]. If these effects are oxytocin-dependent, we would expect aPVN oxytocin neurons to have stronger changes in activity during avoidance and vigilance than pPVN oxytocin neurons. This hypothesis could be tested using calcium imaging of oxytocin neurons, as recently done by several investigators [55, 56].

### Oxytocin produced in the posterior PVN promotes social approach

Our findings support the view that oxytocin neurons in the pPVN facilitate social approach. Although we did not determine whether our manipulations in California mice targeted magno- or parvocellular oxytocin neurons, others have shown that in *M. musculus* and Wistar rats, parvocellular oxytocin neurons are concentrated in the pPVN [31, 39]. Notably, PVN oxytocin neurons send projections to both the NAc and VTA [25, 26, 57, 58], regions where oxytocin receptor activation promotes social approach. For example, oxytocin receptor antagonist in the NAc impairs social bonding in prairie voles [19], social approach in California mice [18], and socially conditioned place preferences [30]. The activation of oxytocin receptors in VTA also has strong effects on social motivation [59, 60]. Interestingly, there is species variability in oxytocin receptor expression in the NAc. In mice, only D1R medium spiny neurons and interneurons express oxytocin receptors [21] while in prairie voles both D1R and D2R medium spiny neurons express oxytocin receptor [61]. These patterns may contribute to species differences in behavior, such as pair bonding.

Recordings in Oxt^Cre^ mice of PVN oxytocin neurons projecting to the NAc found that the majority of neurons exhibited parvocellular electrophysiological characteristics [31]. While parvocellular oxytocin neurons are most prevalent in the pPVN, some magnocellular oxytocin neurons are intermixed with them. Tracing studies in C57Bl6/J mice show that while most oxytocin neurons projecting to the VTA are parvocellular, a few are magnocellular [57]. Thus, in addition to magnocellular oxytocin neurons showing similar morpho-electrical properties to parvocellular oxytocin neurons in the PVN [29], there may be some shared functional properties as well. It’s possible that decreased oxytocin signaling from pPVN neurons may contribute to stress-induced changes in social approach. Future studies could test this via viral overexpression of oxytocin or via optogenetic excitation of pPVN oxytocin neurons.

### Electrophysiological distinctions and implications

The physiological differences we observed in Oxt^Cre^ mice between aPVN and pPVN oxytocin neurons support the idea that these populations are functionally distinct. Anterior PVN oxytocin neurons shared several key electrophysiological properties with oxytocin neurons in the BNST, including significantly higher sPSC frequency and spike frequency. Both aPVN and BNST oxytocin neurons exhibited a longer latency to spike after current injections, resembling the A-type potassium current that is characteristic of magnocellular oxytocin neurons [28]. Posterior PVN oxytocin neurons had shorter latencies to spike, resembling the T-type potassium currents observed in parvocellular oxytocin neurons [39]. In *M. musculus*, there have been several reports that magnocellular oxytocin neurons dominate in the aPVN, while parvocellular are more prevalent in the pPVN [31]. Our electrophysiological results are consistent with these reports.

There were intriguing physiological similarities between aPVN and BNST oxytocin neurons. Our morpholino data indicate that aPVN oxytocin neurons, like BNST oxytocin neurons [20], drive stress-induced social avoidance and vigilance. An interesting possibility is that BNST and aPVN oxytocin neurons may share a cellular origin. Although the developmental origins of oxytocin neurons in the BNST are unknown, previous work indicates that BNST vasopressin neurons in rats are formed on embryonic day 12 (E12) and E13, while non-vasopressin cells originate between E14-E16 [62]. Similarly, the majority of magnocellular neurons (which includes oxytocin and vasopressin neurons) are generated between E12 and E13 in rats [63]. This raises the possibility that oxytocin neurons outside of the hypothalamus, often referred to as the accessory nuclei [28], could originate within the hypothalamus and migrate to their final location in the adult brain. There is precedent for this concept as gonadotropin-releasing hormone neurons originate in the developing olfactory epithelium and migrate through the basal forebrain until reaching the hypothalamus [64]. It is important to note that BNST oxytocin neurons do not fully mimic the electrophysiological profile of the aPVN oxytocin neurons. This suggests that extrinsic factors such as cellular inputs or adjacent glial cells may impact the electrophysiological properties of BNST oxytocin neurons. Overall, our results showing similarities between aPVN and BNST oxytocin neurons suggest that future studies exploring the origin of extra-hypothalamic oxytocin neurons are warranted.

While we did not use tracing techniques to identify magnocellular oxytocin neurons, our observed physiological differences between aPVN and pPVN oxytocin neurons were largely consistent with previously identified characteristics of magno- and parvocellular oxytocin neurons [27, 39]. However, there is growing evidence that a binary classification of oxytocin neurons may not fully capture observed complexity. Rigorous morpho-electric profiling revealed some overlap in physiological profiles in magno- and parvocellular neurons [29]. An unsupervised clustering analysis of morpho-electric properties used action potential kinetics, dendritic complexity, soma size, and intrinsic excitability. This analysis created several clusters that included both magno- and parvocellular neurons. For example, magno- and parvocellular oxytocin neurons found in the pPVN were found to have similar characteristics. Additionally, along the anterior-posterior axis of the PVN, electrophysiological and morphological traits gradually shifted among magnocellular oxytocin neurons. Interestingly there is also evidence for coordination between magno- and parvocellular oxytocin neurons, as some parvocellular oxytocin neurons can modulate the activity of magnocellular oxytocin neurons in the PVN [39]. Together, these findings suggest that the form and function of oxytocin neurons changes across the anterior-posterior axis of the PVN.

## Conclusions

We observed that physiologically and functionally, aPVN oxytocin neurons differed from pPVN oxytocin neurons. In several dimensions, aPVN oxytocin neurons were more similar to BNST oxytocin neurons than pPVN oxytocin neurons, raising important questions about the cellular origin and development of these neurons. Previous tracing studies have focused primarily on differentiating between magnocellular and parvocellular neurons, with less attention paid to the relative location of these cells along the anterior-posterior axis. While recent morpho-electrical analyses suggested that this gradient is important for the physiological properties of PVN oxytocin neurons, it was unclear if this translated into functional differences. Our results suggest that indeed there are important functional differences between oxytocin produced in the anterior versus posterior PVN. Further study is needed to more fully characterize the projections of these neurons and to determine whether genetic markers can be identified that can predict the spatial location of oxytocin neurons within the hypothalamus.

## Supplementary information

Supplementary Material

## Data Availability

Experimental data are available by request to BCT.
