# Supplementary material for: Oxytocin neurons in the anterior and posterior paraventricular nucleus have distinct behavioral functions and electrophysiological profiles
Source: Neuropsychopharmacology. 2026 Jan 17;51(5):946–55. doi: 10.1038/s41386-026-02352-y (PMC13003577; doi:10.1038/s41386-026-02352-y)
Supplement: Supplementary file 1 — Supplementary Material [file 41386_2026_2352_MOESM1_ESM.docx]

**Supplementary methods for “Oxytocin neurons in the anterior and posterior paraventricular nucleus have distinct behavioral functions and electrophysiological profiles**”

*Animals*

California mice were housed in clear propylene cages with wire bar tops in groups of same-sex groups of 2-4. The cages were bedded with Sani-Chip (Harlan Laboratories, Indianapolis, IN, USA), Enviro-Dri (Eco-bedding, Fibercore, Cleveland, OH, USA), and Nestlets (Ancare, Bellmore, NY, USA). All procedures followed NIH guidelines and were approved by UC Davis and Michigan State University Institutional Animal Care and Use Committees.

*Electrophysiology*

*Ex vivo* whole cell slice electrophysiology was conducted in *Oxt*^Cre^/Rosa^L10eGFP^ mice aged 7-10 weeks. All solutions were bubbled with 95% O_2_ -5% CO_2_ throughout the procedure. Mice were anesthetized with isofluorane and transcardially perfused with ice cold sucrose artificial cerebrospinal fluid (aCSF) (234 mM sucrose, 26 mM NaHCO_3_, 11 mM D-glucose, 10 mM MgSO_4_, 2.5 mM KCl, 1.25 mM NaH_2_PO_4_, 0.5 mM CaCl_2_). Brains were rapidly removed, blocked, and sectioned into 250 μM coronal sections containing the brain region(s) of interest using a vibratome (Leica VT1200S R). Brain slices were transferred to an incubation chamber containing saline aCSF (126 mM NaCl, 26 mM NaHCO_3_, 10 mM glucose, 2.5 KCl, 2 mM MgCl_2_, 1.25 mM NaH_2_PO_4_) held at 37° C for 30 min, then at room temperature until used for recordings. Recordings were made from slices held in a submersion chamber perfused with saline aCSF (2 mL/min) held at 32 °C with an inline heater (Warner Instruments). Borosilicate glass electrodes (3–6MΩ) were filled with a potassium gluconate internal solution (115 mM potassium gluconate, 20 mM KCl, 10 mM phosphocreatine-di(Tris), 2 mM Mg-ATP, 1.5 mM MgCl_2_, 0.5 mM Na 3 -GTP; pH 7.2; 285–295 mOsm). GFP-positive cells were visualized using an Olympus BX51WI microscope using DIC infrared and epifluorescent illumination. Whole-cell patch-clamp recordings were made from cells using a Multiclamp 700B amplifier and Digidata 1440A digitizer (Molecular Devices) and whole-cell junction potential was not corrected. Recordings were sampled (10 kHz), filtered (10 kHz), and digitally stored. Membrane capacitance, membrane resistance, and access resistance were automatically calculated by pClamp 10 software (Molecular Devices). Cells with access resistance greater than 40 mΩ were omitted from analysis. Resting membrane potential was measured by the Multiclamp without injecting current (I=0). Spontaneous PSC event frequency and amplitude were determined from gap free voltage clamp recording and analyzed using MiniAnalysis software (Synaptosoft, Inc.) and Clampfit Minis Search software (Molecular Devices). Excitability of neurons was measured by increasing depolarizing steps (0 to 100 pA, Δ25 pA steps, 500 ms) with 30 s step intervals. Presence of a transient outward rectification, which is found in magnocelluar oxytocin neurons but not parvocellular oxytocin neurons was used to differentiate the neuron types. To find this transient outward rectification, a minimum current to reach ~-100 mV (200 ms) was applied followed by increasing depolarizing steps (200 ms, Δ25 pA) with a 2 s sweep interval.

*Tissue collection for histology*

For California mice, one day following the social interaction test, animals were euthanized using isoflurane, then perfused with 4% paraformaldehyde (PFA). The brains were collected, stored in 4% PFA overnight, then switched to 30% sucrose solution for a minimum of 48 hours. Following sucrose treatment, the brains were flash frozen with dry ice then immediately stored in -80° C. Frozen brains were sliced coronally with a cryostat (Leica) at -20° C at 40 mm and stored in cryoprotectant. For *Oxt*^Cre^ validation studies, mice were deeply anesthetized and transcardically perfused with ice cold PBS following by 10% formalin. Brains were fixed overnight with 10% formalin then stored in 30% sucrose until sectioned to 35 mm using a freezing microtome (Leica SM2010R).

*Immunohistochemistry*

For California mice coronal slices at 120 µm intervals containing the PVN were selected. After removal from storage in cryoprotectant, the slices were washed twice for 5 minutes in phosphate-buffered saline (PBS) before being blocked with 10% normal goat serum. Then, the tissue was then placed in mouse anti-oxytocin primary antibody (1:2000; Millipore Sigma PS38) in 2% normal goat serum for 24 hours at 4° C. This primary antibody has been validated for specificity in California mice (Steinman et al., 2015). After primary antibody incubation, the slices were washed three times for 5 minutes in PBS to remove all unbound antibodies before incubating with secondary antibody goat anti-mouse Alexa-Fluor 555 (1:500, ThermoFisher A21424) in 2% normal goat serum for 2 hours at room temperature. After another 5-minute wash in PBS, sections were mounted onto glass slides and coverslipped with mounting medium (Vectashield). For oxytocin staining of *M. musculus* in Figure 1, a wild-type perfused mouse brain was used, and the process was repeated, but using rabbit anti-oxytocin (1:2500, Millipore AB911) and goat anti-rabbit 555 (1:500, Invitrogen A21429).

For *Oxt*^Cre^ mice, brain slices were washed with 0.3% Tween20 in PBS three times for 10 minutes, then blocked with 10% normal donkey serum before incubating in primary antibodies goat anti-GFP (1:4000; Abcam, ab5450) and mouse anti-oxytocin (1:1000; Millipore Sigma PS38) in 10% normal donkey serum and 0.6% TritionX100 in PBS overnight. After primary antibody incubation, the slices were washed three times for 5 minutes in PBS to remove all unbound antibodies before incubating with secondary antibody 1:500 secondaries donkey anti-goat AF488 and anti-mouse Cy3 in 1% normal donkey serum and 0.3% Tween20 in PBS. Sections were mounted onto glass slides and cover slipped with DPX mounting media (Sigma). Fluorescent images were taken using a Nikon Eclipse Ni-U Upright Fluorescent Microscope.

**Supplementary Figures**


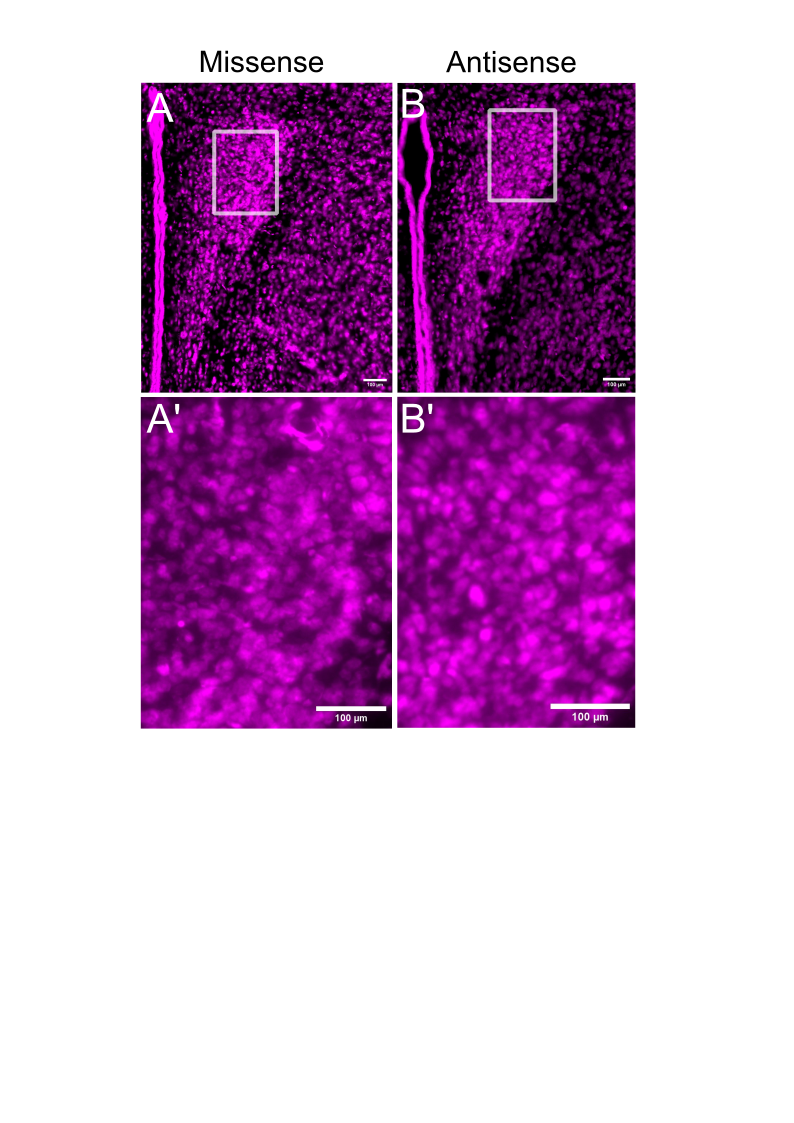


**Figure S1.** Nissl stains indicate no evidence of cytotoxicity via antisense morpholino treatment in the PVN, consistent with Duque-Wilckens et al., 2020.


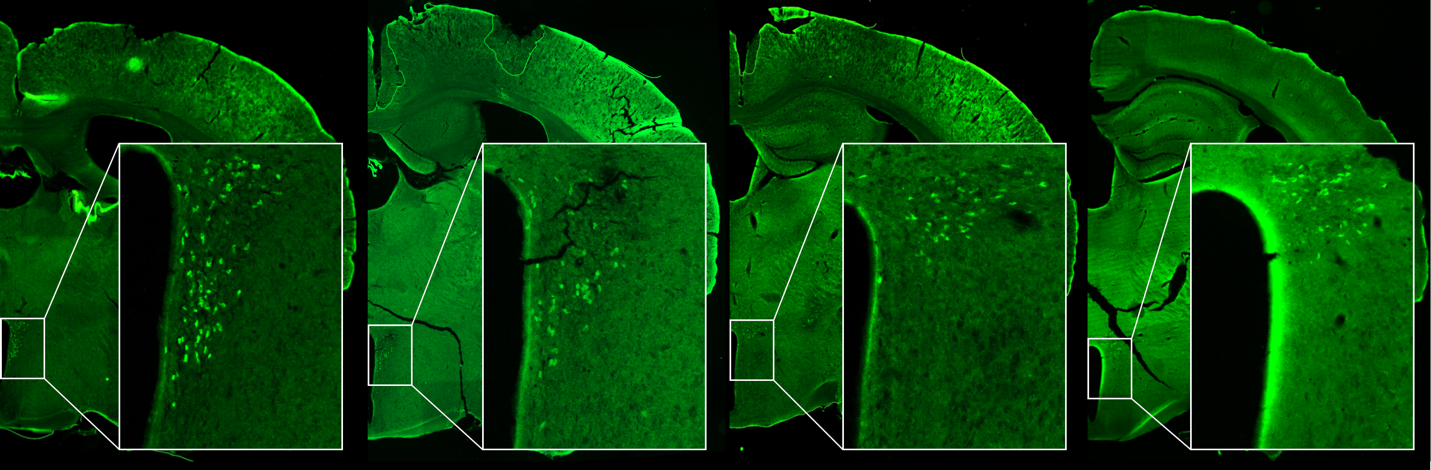


**Figure S2.** Representative 10x images showing distribution of GFP-positive oxytocin neurons across the anterior-posterior axis of the paraventricular nucleus from Oxt^Cre^/Rosa^L10eGFP^ (*Mus Musculus*) mice.


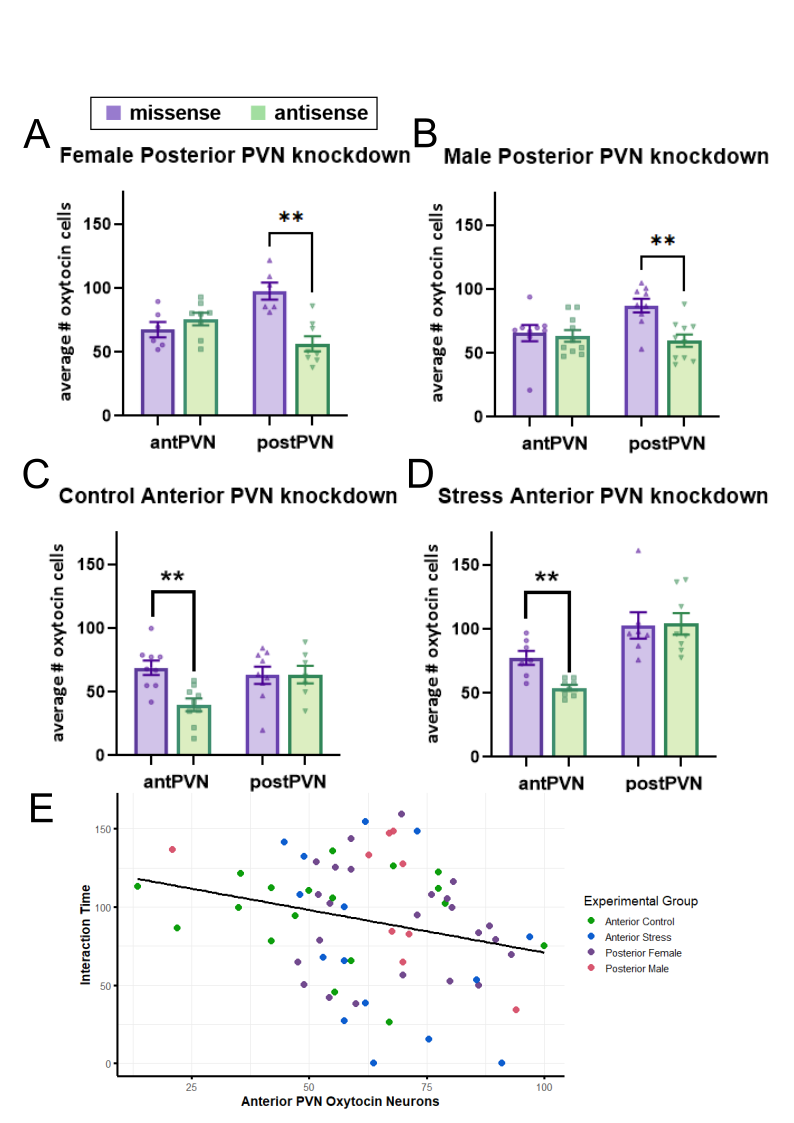


**Figure S3.** Knockdown of oxytocin impacted all experimental groups. Antisense morpholino treatment reduced oxytocin neurons in the targeted PVN subregion in (A) female, (B) male, (C) control female, and (D) stressed female mice. ** p < 0.001 vs missense.


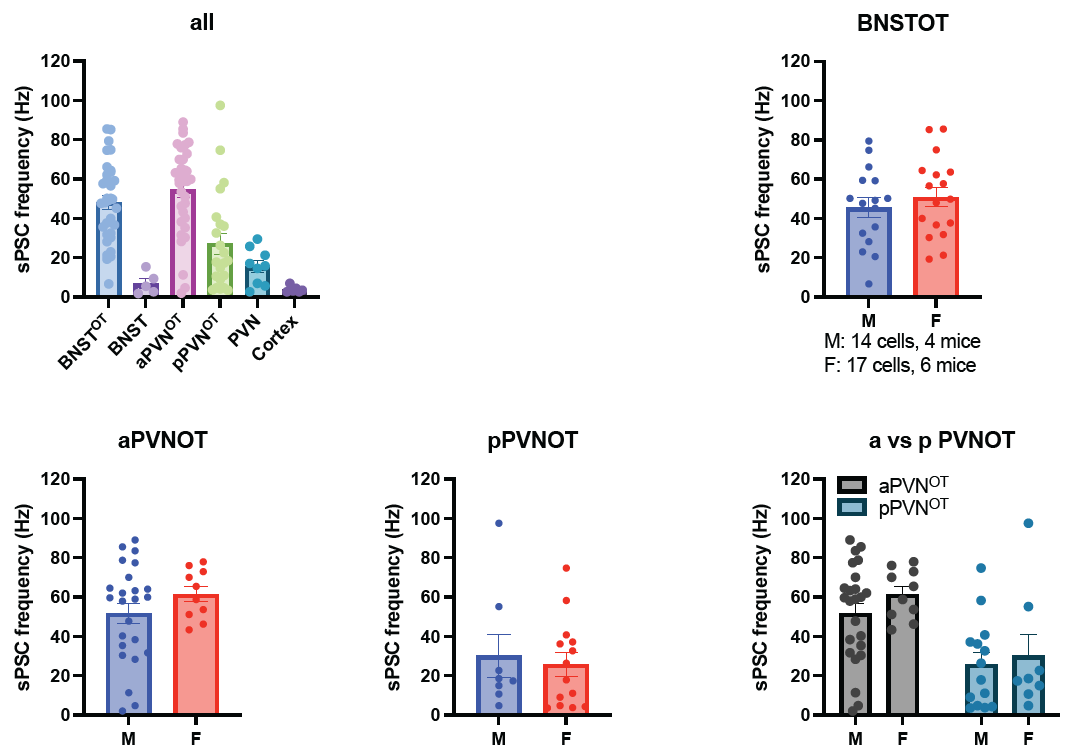


**Figure S4**. Data for spontaneous post-synaptic currents (sPSC) graphed for all cell types collapsed by sex (same as Fig 4J) and plotted by sex for bed nucleus of the stria terminalis (BNST), anterior PVN oxytocin (aPVNOT), posterior PVN oxytocin (pPVNOT) and with anterior and poster PVNOT neurons plotted together on the same graph.

**Table S1.** The percent change of oxytocin immunoreactive cell counts in the targeted PVN subregion across experimental groups.


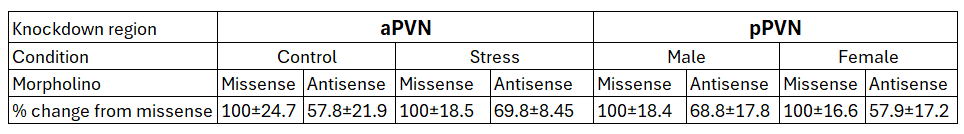


**Table S2.** Two-way ANOVA testing the effects of morpholino treatment and stress on social interaction.


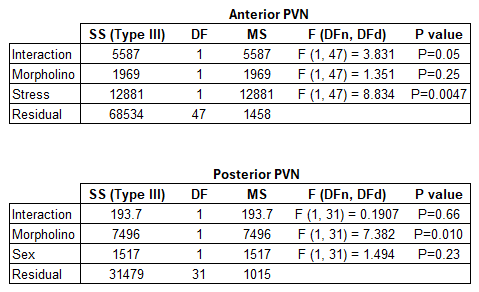

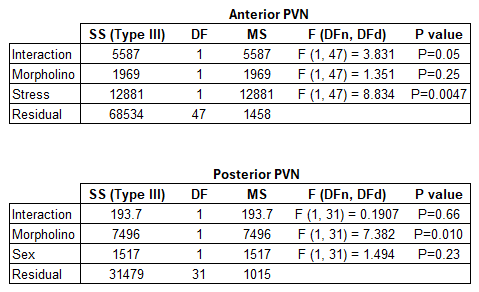


**Table S3.** Two-way ANOVA testing the effects of morpholino treatment and sex on social interaction.
